# Multi-omics integration reveals Chr1 associated QTL mediating backfat thickness in pigs

**DOI:** 10.1186/s40104-025-01254-1

**Published:** 2025-10-20

**Authors:** Naibiao Yu, Dengshuai Cui, Chenyu Li, Siyu Yang, Chuanmin Qiao, Lei Xie

**Affiliations:** 1 National Key Laboratory for Swine Genetic Improvement and Germplasm Innovation, Ministry of Science and Technology of China, Jiangxi Agricultural University, Nanchang, 330045 China; 2 Sanya Institute, Hainan Academy of Agricultural Sciences, Sanya, 572025 China

**Keywords:** Backfat thickness, Deep learning, GWAS, Multi-omics, Pig

## Abstract

**Background:**

Backfat thickness (BFT) is a vital economic trait in pigs, reflecting subcutaneous fat levels that affect meat quality and production efficiency. As a complex trait shaped by multiple genetic factors, BFT has been studied using genome-wide association studies (GWAS) and linkage analyses to locate fat-related quantitative trait loci (QTLs), but pinpointing causal variants and genes is hindered by linkage disequilibrium and limited regulatory data. This study aimed to dissect the QTLs affecting BFT on *Sus scrofa* chromosome 1 (SSC1), elucidating regulatory variants, effector genes, and the cell types involved.

**Results:**

Using whole-genome genotyping data from 3,578 pigs and phenotypic data for five BFT traits, we identified a 630.6 kb QTL on SSC1 significantly associated with these traits via GWAS and fine-mapping, pinpointing 34 candidate causal variants. Using deep convolutional neural networks to predict regulatory activity from sequence data integrated with detailed pig epigenetic profiles, we identified five SNPs potentially affecting enhancer activity in specific tissues. Notably, rs342950505 (SSC1:161,123,588) influences weak enhancer activity across multiple tissues, including the brain. High-throughput chromosome conformation capture (Hi-C) analysis identified that rs342950505 interacts with eight genes. Chromatin state annotations confirmed enhancer activity at this QTL in the cerebellum. Leveraging these insights, single-cell ATAC-seq revealed a chromatin accessibility peak encompassing rs342950505 that regulates *PMAIP1* expression in inhibitory neurons via enhancer-mediated mechanisms, with an adjacent peak modulating *CCBE1* expression in neuroblasts and granule cells. Transcriptome-wide association studies (TWAS) confirmed *PMAIP1*’s role in the hypothalamus, and Mendelian randomization (MR) validated *PMAIP1* and *CCBE1* as key brain expression quantitative trait locus (eQTL) effectors. We propose that the variant rs342950505, located within a regulatory peak, modulates *PMAIP1* expression in inhibitory neurons, potentially influencing energy homeostasis via hypothalamic regulation. Similarly, *CCBE1* may contribute to this process.

**Conclusions:**

Our results, through systematic dissection of pleiotropic BFT-associated loci, provide a framework to elucidate regulatory mechanisms of complex traits, offering insights into polygenic control through lipid metabolism and neural signaling pathways.

**Supplementary Information:**

The online version contains supplementary material available at 10.1186/s40104-025-01254-1.

## Background

Subcutaneous fat deposition, a key trait in swine production, influences meat quality, animal health, and feed efficiency [1, 2]. Moderate fat improves palatability, while excessive deposition diminishes lean meat yield—an important goal in modern commercial breeding. Backfat thickness (BFT), a major indicator of fat deposition [3] with modest heritability (0.2–0.6) [4, 5], can be measured in vivo via ultrasonography or at slaughter, allowing for genetic improvement. Dissecting its genetic basis could optimize leanness and bolster local breed conservation, despite significant challenges [6].

So far, linkage analyses and genome-wide association studies (GWAS) have identified a large number of significant quantitative trait loci (QTLs) for fat deposition and related traits in the pig QTL database (https://www.animalgenome.org/QTLdb) [7], revealing a polygenic architecture with heritability spread across many small-effect, mostly noncoding loci. Yet, only a few candidate causal genes—*IGF2* [8, 9], *MC4R* [10, 11], and *LEPR* [12–14]—have been confirmed for BFT and fat traits. Our previous study identified a 159–162 Mb region on *Sus scrofa* chromosome 1 (SSC1) tied to subcutaneous fat deposition across multiple sites in commercial pigs, which harbor independent QTLs [15]. Although the *MC4R* p.Asp298Asn [16, 17] variant lies within this span, fine-mapping suggests that it is not the primary causal locus, implying undiscovered genes and variants. Reinforcing this, Boshove et al. [18] identified a small, complex region spanning 5 Mb on SSC1 that contains four independent QTLs influencing growth rate and BFT. We thus hypothesize that additional QTLs on SSC1 influence BFT in commercial pigs, yet their underlying genetic architecture, particularly the “variant-to-gene” and “variant-to-function” relationships, remains elusive.

Despite this hypothesis, limited single-population GWAS power, tissue-specific regulatory data, and linkage disequilibrium (LD) make it difficult to identify causative variants for BFT. Fortunately, recent advances in multi-GWAS and meta-GWAS have enhanced detection power [19], tools like FINEMAP have narrowed QTLs, linking *FAM13A* to human fat distribution [20]. Similarly, SuSiEx has improved cross-population fine-mapping, as evidenced by its application to schizophrenia loci in the Taiwan Biobank and UK Biobank [21]. Transcriptome-wide association studies (TWAS) and molQTL colocalization identified *SNX10* as a causal gene in sexually dimorphic adipose distribution [22]. Additionally, the sequence-based deep convolutional neural network (CNN) *Basenji* integrates multi-tissue or single-cell epigenomic data to uncover regulatory mechanisms of complex traits, successfully identifying causal variants for traits such as metabolic obesity [23], kidney failure [24], CCDDs [25], hyperglycemia, and bone mineral density [26]. These approaches provide a robust framework for dissecting complex trait architectures.

Building on these advancements, we aimed to elucidate the genetic architecture of BFT on SSC1, encompassing regulatory variants, effector genes, and relevant cell types, across anatomical regions and their regulation of fat deposition in pigs. By integrating multi-omics approaches (including GWAS, a *Basenji* deep CNN model, high-throughput chromosome conformation capture (Hi-C), tissue chromatin state annotations, single-cell ATAC sequencing (scATAC-seq), Mendelian randomization (MR), TWAS, colocalization, and MAGMA), we sought to resolve this complexity and address existing challenges. This integrative strategy improves QTL resolution, prioritizes candidate variants, and clarifies their roles in fat deposition, advancing insight into the genetic basis of this economically important trait in pigs.

## Materials and methods

### Phenotype

In this study, we collected phenotypic data from 3,578 experimental pigs. A total of 2,012 initial experimental pigs were divided into four genetic groups: 265 Landrace (LR, 95 sows and 170 barrows), 698 Yorkshire (YK, 435 sows and 263 barrows), 689 Landrace $\times$ Yorkshire crossbred (LY, 402 sows and 287 barrows), and 258 Duroc $\times$ Landrace $\times$ Yorkshire crossbred (DLY, 115 sows and 143 barrows) [27, 28]. Due to the limited number of DLY individuals in the initial sample, additional phenotypic data was collected for 1,566 DLY crossbred pigs (Duroc $\times$ Landrace $\times$ Yorkshire) in the following phase of the study in Guangdong Province, China.

Backfat thickness was measured using standard protocols at four anatomical sites on the left side of the carcass: shoulder backfat depth (SBD), 6^th^–7^th^ rib backfat depth (RBD), waist backfat depth (WBD), and hip backfat depth (HBD). The mean backfat depth (MBD) was calculated using the following formula: $\text{MBD} = (\text{SBD }+\text{ RBD }+\text{ WBD }+\text{ HBD}) / 4.$

### Genotype

The genotype data for the 2,012 samples in the initial phase were obtained by genotyping using the CC1 PorcineSNP50 BeadChip (51,368 SNPs), followed by imputation using a reference haplotype library constructed by our laboratory [29]. The imputation process and quality control procedures are described in detail in a previous study [15]. After imputation and quality control, 26,773,790 SNPs were identified.

In the second phase, 1,566 DLY population samples were collected, and ear tissue was obtained from each individual for DNA extraction. Genotyping was then performed using 10X coverage DNA sequencing with second-generation sequencing. Post-sequencing data were processed with Fastp v0.23.0 [30], which filtered paired-end reads by removing sequences containing $\ge$ 10% missing bases (“N”) or with a quality score $\le$ 20 for $\ge$ 50% bases. Reads shorter than 75 bp were discarded. The clean reads were then aligned to the *Sus scrofa* reference genome 11.1 [31] using BWA v0.7.17 [32] (BWA-MEM algorithm, default parameters). BAM files of mapped reads were sorted by genome position and marked for PCR duplication using Samtools v1.9 [33]. The gvcf files were generated using the official GATK v4.1.4.1 [34] pipeline, which included the BaseRecalibrator, ApplyBQSR, and HaplotypeCaller. Joint genotyping of all gvcf files was performed using GLNexus [35] to produce the final VCF file. Variants were filtered with PLINK v2.0.0-a.5.22 [36] according to the following specific criteria: SNPs with a minor allele frequency (MAF) $<$ 1%, more than 80% missing genotypes, or multi-allelic sites were excluded. Variants with half calls in the VCF file were treated as missing genotypes. Following these steps, the number of remaining SNPs was determined to be 25,809,125 in the additional DLY population.

### Single and combine two population GWAS (multi-GWAS)

Single and combined two population single-locus association analyses were conducted using the GEMMA software (v0.98.1) [37] with a linear mixed model (LMM) that accounts for the SNP-based population structure and relatedness between individuals. The mixed linear model is,

$${\varvec{y}}={\varvec{X}}{\varvec{b}}+{\varvec{Z}}{\varvec{u}}+{\varvec{e}}$$

where ${\varvec{y}}$ is the phenotypic vector, ${\varvec{X}}$ is the fixed effect indicator matrix including sex, populations and slaughter batches, ${\varvec{b}}$ is the corresponding estimations of fixed effect estimates, ${\varvec{Z}}$ is the design matrix of multiple markers, ${\varvec{u}}$ is the SNP substitution effect, and ${\varvec{e}}$ is the vector of random residual effects. It is assumed that ${\varvec{u}}$ is derived from a multivariate normal distribution ${\varvec{u}}$ ~ $N$ (0, ${\varvec{A}}{\sigma }_{u}^{2}$), with a kinship matrix GRM for ${\varvec{A}}$ and ${\sigma }_{u}^{2}$ is the additive genetic variance, and the residuals ${\varvec{e}}$ are derived from a normal distribution ${\varvec{e}}$ ~ $N$ (0, ${\varvec{R}}{\sigma }_{e}^{2}$). To account for multiple comparisons, applying Bonferroni corrections (0.05 divided by the number of SNPs) would lead to an overly stringent threshold due to the high correlation among many SNPs. Instead, Pe’er et al. [38] and Johnson et al. [39] proposed a genome-wide significance threshold of 5 $\times$ 10^−8^. Additionally, a chromosome-wide significance threshold of 1 $\times$ 10^−6^ was used as a suggestive significance threshold [40, 41].

Genomic heritability of BFT phenotypes and the proportion of phenotypic variance explained by significant SNPs were estimated using GCTA [42]. Genetic correlations among phenotypes were calculated to assess shared genetic architecture and pleiotropic effects.

### Meta-analysis of GWAS (meta-GWAS)

In the initial population of 2,012 pigs, significant GWAS signals were primarily found for the RBD, WBD, and MBD. Similarly, in the additional DLY population, significant GWAS signals were observed for the RBD, WBD, HBD, and MBD. Therefore, to increase the detection of significant GWAS signals, the focus was placed on backfat thickness traits at four key anatomical sites as well as mean backfat thickness in the subsequent meta-analysis. Meta-analysis was performed using the inverse-variance fixed-effects method in METAL with genomic control adjustment [43].

### Pigs LocusZoom

LocusZoom plots were generated for swine by integrating multiple datasets with the *Sscrofa*11.1 reference genome [44]. Gene annotations were extracted from the *Sscrofa*11.1 GFF file (Ensembl). SNP position files were then constructed by merging these annotations with PLINK files from two populations. A LocusZoom database tailored to *Sscrofa*11.1 was built for each population using these combined datasets. Finally, linkage disequilibrium (LD) for the relevant loci was calculated using summary statistics and genotype binary files, enabling the visualization of LocusZoom plots.

### Statistical fine mapping

To narrow the intervals of the identified loci, this study employed FINEMAP v1.4 [45] for genetic fine-mapping using a shotgun stochastic search algorithm. The detailed procedures are listed below: First, heuristic fine-mapping approaches were used to reduce linkage disequilibrium (LD) by 0.2 for the most significant loci across the merged populations [46]. Next, the maximum and minimum positions were determined based on the chromosomal intervals of each trait and were used as baselines to define the target fine-mapping intervals. The GWAS summary files of each trait were then screened to exclude loci with a *P*-value threshold below 1 $\times$ 10^–2^. Finally, the processed files were used as inputs for the FINEMAP fine-mapping process. The parameters for the FINEMAP analysis were set with a 95% confidence interval, allowing for up to one to four causal variants per locus. The fine-mapping results for each locus included the Bayes factor and posterior probability of each variant being pathogenic. This method identified candidate causal variants by modeling the LD structure of the locus and the strength of association (Z-score). To ensure data integrity, a customized pre-processing workflow was designed and implemented prior to the formal fine-mapping stage.

Subsequently, we performed multi-ancestry fine-mapping on the merged population's reference panel, incorporating its LD structure, using SuSiEx [21]. For this analysis, GWAS summary statistics from a single-marker GWAS conducted separately for each population were utilized. Fine-mapping was performed within the interval SSC1:160,883,673–161,514,225, with a 95% confidence interval. Each trait was fine-mapped in every population and the results were subsequently integrated across all traits.

### Gene-based association study

To identify genes associated with BFT traits, genome-wide gene-based association study was conducted to detect genes strongly associated with BFT across different populations. A gene-based association study was performed using MAGMA v1.10b [47] on the meta-GWAS summary results, which included 32,130 genes for analysis and leveraged a high-quality thousand-pig reference genome constructed by our laboratory [29]. Finally, the analysis comprised 29,712 genes. The significance criterion was 3.36 $\times$ 10^–7^, calculated by dividing 0.01 by the total number of genes.

### High-throughput chromosome conformation capture (Hi-C) data analysis

Hi-C sequencing was performed on subcutaneous adipose tissue (backfat) from a single pig, as part of a previously described population [48]. The in situ Hi-C library was constructed following the method described by Rao et al. [49], with the addition of a custom MboI restriction enzyme recognition site. Raw Hi-C reads were quality-filtered using FastQC, and reads shorter than 100 bp were discarded. The clean data were then processed using the Juicer pipeline [50] with default parameters. Contact maps were generated at 25 kb and 5 kb resolutions, both of which passed the Juicer resolution prediction [50]. Topologically associating domains (TADs) were identified at 25 kb resolution using the HiCExplorer algorithm (v3.5.1) [51], while chromatin loops were detected at 5 kb resolution using Mustache (v1.0.1) [52]. Finally, the genomic tracks were visualized using pyGenomeTracks [53].

### Single-cell ATAC sequencing (scATAC-seq) analysis

In parallel, brain single-cell ATAC sequencing (scATAC-seq) data were obtained from a previously published dataset generated by our laboratory [54]. This dataset comprised six Bamaxiang pigs sampled at four key postnatal developmental stages: 30 d (*n *= 2, preweaning), 42 d ($$n\,$$$$=$$ 2, postweaning), 150 d ($$n\,$$$$=$$ 1, rapid growth), and 730 d (*n* = 1, adulthood).

### Molecular QTL–GWAS integration

After fine-mapping, the QTL interval was successfully narrowed. However, further investigation is required to clarify the downstream effects of relevant variants on various fat deposition-associated tissues, cell types, and molecular phenotypes, including gene expression, splicing, protein expression, methylation, and histone acetylation. To identify candidate causal mutations and their associated genes, we used additional data sources. Specifically, we collected molecular QTL data from PigGTEx [55], including summary statistics for eQTL, eeQTL, enQTL, lncQTL, and sQTL, collectively termed as molecular quantitative trait loci (molQTL).

### Colocalization analysis

To investigate whether the genetic association between gene expression in a specific tissue and fat deposition at various sites arose from identical causal variants, we integrated GWAS with molQTLs. We performed colocalization analyses using the coloc.abf() function from the coloc R package (v5.2.3) [56] with default prior settings to detect colocalized SNPs within a predefined range of lead variants established prior to fine-mapping. The H4 test statistic measures the likelihood of a common causal variation between fat deposition and gene expression in specific tissues. The H4 statistic is interpreted as follows: H4 $>$ 0.7 shows significant evidence of colocalization, H4 between 0.5–0.7 suggests poor evidence of colocalization, and H4 $<$ 0.5 implies no colocalization [57].

### Transcriptome-wide association study (TWAS)

For the TWAS analysis of the merged populations, we used the following datasets: (1) predicted molQTLs models for 20 tissues and their corresponding covariate files from PigGTEx and (2) GWAS summary statistics for the merged populations. Using SPrediXcan v0.7.5 [58], we estimated the association between gene expression in these tissues and fat deposition at various anatomical locations. This analysis identified tissues and genes associated with fat deposition across different loci.

### Mendelian randomization (MR)

We conducted a bidirectional two-sample MR analysis [59] using TwoSampleMR (v0.6.5) in R (v4.3.2) to assess causal links between gene expression and five distinct backfat thickness traits. Multi-tissue eQTL data from PigGTEx [55] (*P *$<\,$ 0.05) and GWAS summary statistics for backfat thickness from this study (*P *$<\,$ 15 $\times$ 10^–4^) were derived from independent populations. Forward MR used GWAS as exposure and eQTL as outcome; reverse MR inverted the roles. Both applied inverse-variance weighted (IVW), MR-Egger [60], weighted median [61], simple mode, and weighted mode. Sensitivity tests (MR-Egger intercept, heterogeneity; *P *$<$ 0.05) evaluated pleiotropy and heterogeneity. Forest plots of causal estimates were generated using the *forest* function in the forestploter package (v1.1.2) in R.

### Key tissues linked to back subcutaneous fat deposition

To identify tissues potentially influencing fat deposition across different anatomical sites, we collected chromatin state data for 14 porcine tissues representing 15 distinct chromatin states. The tissues analyzed included adipose tissue, cecum, cerebellum, colon, cerebral cortex, duodenum, hypothalamus, ileum, jejunum, liver, lungs, muscle, spleen, and stomach. Chromatin states encompass promoters (TssA, TssAHet, TssBiv), TSS-proximal transcribed regions (TxFlnk, TxFlnkWk, TxFlnkHet), enhancers (EnhA, EnhAMe, EnhAWk, EnhAHet, EnhPois), repressor regions (Repr, ReprWk), quiescent regions (Qui), and ATAC islands, which are accessible, but do not overlap with any other measured epigenetic marks [62].

Additionally, to pinpoint the candidate causal variants influencing fat deposition in different porcine tissues, we integrated epigenetic data, including ATAC-seq and ChIP-seq. These data were collected from FAANG [63], Swine ENCODE [64], and Ensembl (Table S1).

### Chromatin state segmentation and visualization

Based on the fine-mapping intervals and the chromatin state annotation data, we analyzed the tissues where these intervals were enriched for regulatory regions. These tissues were identified as candidates likely influencing fat deposition at various sites.

### Convolutional neural network (CNN) training and prediction

#### Data processing

We adapted the *Basenji* framework [65], a sequence-based deep CNN, to the swine genome (*Sus scrofa* 11.1, Ensembl). *Basenji* predicts the impact of noncoding variants on regulatory function through epigenetic modifications, leveraging multi-tissue epigenomic data, including ATAC-seq and ChIP-seq for histone modifications. The model mapped 1,344-bp DNA sequences to quantitative chromatin accessibility reads, using a gap-annotated FASTA file from Ensembl as the reference genome. Preprocessing was performed with the basenji_data.py script, configured as follows: --local -p 20 --r 4096 --w 192 --l 1344 --v 0.12 --t 0.12 --stride 192 --stride_test 192 --crop 576. Here, -p 20 enabled 20 parallel processes, --l 1344 set the sequence length to 1,344 bp, --r 4096 defined the resolution, and --crop 576 trimmed sequences for input consistency, --v 0.12 reserved 12% of sequences for validation, and --t 0.12 held out 12% of sequences for testing [24].

#### Training

Training was conducted using the basenji_train.py script on an NVIDIA A300 GPU, with parameters adapted from Basset defaults [66]. The architecture began with a convolutional layer (288 filters, 17-bp filter size, 3-bp max pooling), followed by a six-block convolutional tower with filters increasing from 288 to 512 (5-bp filter size, 2-bp max pooling per block). A subsequent layer (256 filters, 1-bp filter size) fed into a fully connected layer (768 units, 0.2 dropout), ending with an 11-unit output layer using sigmoid activation. We applied GELU activation, batch normalization (momentum 0.90), and data augmentation with reverse complement sequences and 3-bp sequence offsets. Training parameters included a batch size of 64, learning rate of 0.005, momentum of 0.98, and stochastic gradient descent (SGD) with binary cross-entropy (BCE) loss. Early stopping was applied after 12 epochs without improvement, with a minimum of 10 epochs. Model performance was assessed using the basenji_test.py script with default settings.

#### SAD score prediction

To assess allelic effects, we used the trained model to predict SNP Activity Difference (SAD) scores with the basenji_sad.py script, comparing chromatin accessibility between reference and alternate alleles at candidate loci. Variants with the highest absolute SAD values were flagged as putative causal candidates, with their associated tissues being noted. For high-SAD loci, in silico saturation mutagenesis was conducted with the basenji_sat_vcf.py script, testing all four nucleotides at each position. The loss score was the maximum decrease in accessibility when mutating to a non-reference nucleotide, and the gain score represented the maximum increase [66].

## Result

### Descriptive statistics and heritability estimates

In large populations ($n$ $=$ 3,578), descriptive statistics for backfat thickness (BFT) at five traits—SBD, RBD, WBD, HBD, and MBD—showed coefficients of variation from 22.08% to 32.75% (Table 1). Heritability estimates, derived via REML using genetic relationship matrices, were 0.30 $\pm$ 0.03 for SBD and 0.47 $\pm$ 0.03 for MBD (Table 2). Bivariate analysis revealed strong phenotypic correlations (0.56–0.86) and genetic correlations (0.72–0.97) among traits, with the lowest genetic correlation between SBD and HBD (0.72 $\pm$ 0.05) and the highest between MBD and RBD (0.97 $\pm$ 0.01) and WBD (0.95 $\pm$ 0.01) (Table 2). These results indicate shared genetic influences and a partial genetic basis for backfat thickness traits.

**Table 1:** Descriptive statistics for fat deposition related traits

Trait	Unit	Number	Mean (SD)	Minimum	Maximum	CV, %
SBD	cm	3,570	3.13 ± 0.70	1.10	6.80	22.08
RBD	cm	3,571	2.45 ± 0.64	0.80	5.90	25.95
WBD	cm	3,571	2.00 ± 0.62	0.20	4.80	30.81
HBD	cm	3,567	1.67 ± 0.55	0.20	4.70	32.75
MBD	cm	3,570	2.27 ± 0.52	0.84	4.52	22.59

*SBD* Shoulder backfat depth, *RBD* 6^th^–7^th^ rib backfat depth, *WBD* Waist backfat depth, *HBD* Hip backfat depth, *MBD* Mean of backfat depth,* SD* Standard deviation, *CV* Coefficient of variation

**Table 2:** Heritability, genetic and phenotypic correlation coefficients among the fat deposition related traits

Trait	SBD	RBD	WBD	HBD	MBD
SBD	**0.30 ± 0.03**	0.70	0.62	0.56	0.85
RBD	0.87 ± 0.03	**0.37 ± 0.03**	0.66	0.59	0.86
WBD	0.80 ± 0.04	0.89 ± 0.02	**0.37 ± 0.03**	0.60	0.86
HBD	0.72 ± 0.05	0.83 ± 0.04	0.82 ± 0.04	**0.34 ± 0.03**	0.79
MBD	0.91 ± 0.02	0.97 ± 0.01	0.95 ± 0.01	0.89 ± 0.02	**0.47 ± 0.03**

*SBD* Shoulder backfat depth, *RBD* 6^th^–7^th^ rib backfat depth, *WBD* Waist backfat depth, *HBD* Hip backfat depth, *MBD* Mean of backfat depth. Diagonal (bold), and upper and lower triangles are hertabilities and their standard deviations, phenotypic correlation coefficients, genetic correlation coefficients and their standard deviations, respectively

### Dissecting regional backfat genetics with precision GWAS and Fine-mapping

**Fig. 1:**
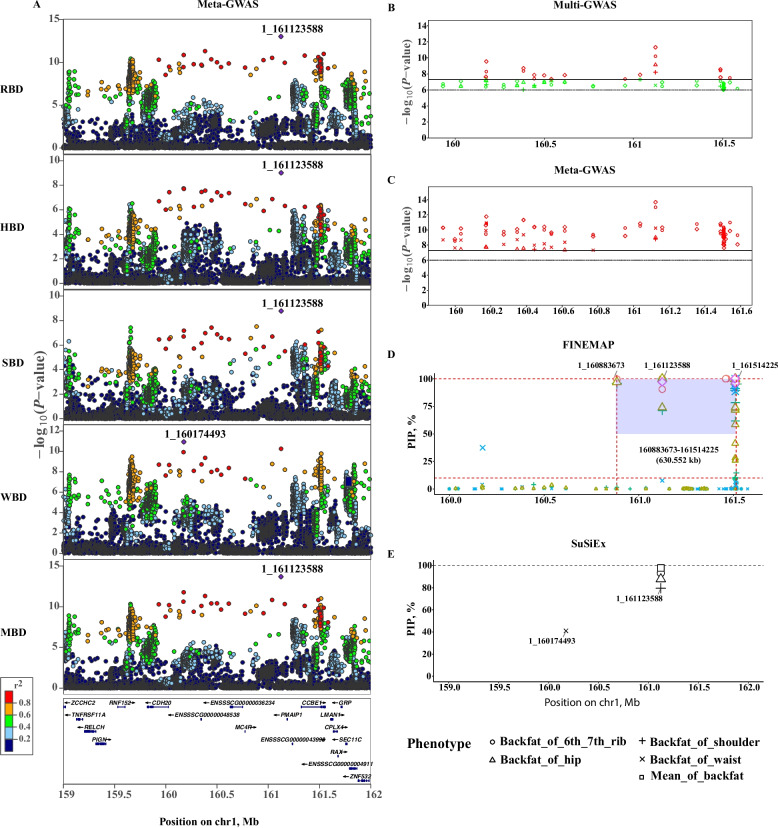
Genome-wide association and fine-mapping results for five backfat thickness traits on SSC1. **A** Manhattan plot of meta-GWAS for five backfat thickness (SBD, shoulder backfat depth; RBD, 6^th^–7^th^ rib backfat depth; WBD, waist backfat depth; HBD, hip backfat depth; MBD, mean backfat depth) on SSC1. **B** Manhattan plot of multi-GWAS (*P* $<$ 5 $\times$ 10^-8^, LD $>$ 0.8). **C** Manhattan plot of meta-GWAS (*P* $<$ 5 $\times$ 10^-8^, LD $>$ 0.8). **D** Fine-mapping Manhattan plot based on multi-GWAS data. **E** Fine-mapping manhattan plot using SuSiEx. 1_161123588 (rs342950505) indicates position 161,123,588 on *Sus scrofa* chromosome 1 (SSC1); other figures use the same format

Building on these heritability and correlation findings, we next investigated the genetic architecture of BFT across five traits (shoulder, 6^th^–7^th^ rib, waist, hip, and mean; SBD, RBD, WBD, HBD, and MBD) in 3,578 pigs using multi-GWAS and meta-GWAS. Multi-GWAS integrated chip-imputed and whole-genome sequencing (WGS) data for comprehensive variant coverage, while meta-GWAS combined summary statistics from two commercial cohorts to enhance statistical power. Applying a genome-wide significance threshold of *P* $$<$$ 5 $$\times$$ 10^–8^, both approaches identified a major QTL on chromosome 1 (SSC1:159–162 Mb) [67]. Multi-GWAS detected 135 significant SNPs (SBD: 1; RBD: 63; WBD: 1; HBD: 6; MBD: 64; Fig. S1), whereas meta-GWAS identified 1,117 significant SNPs (SBD: 5; RBD: 421; WBD: 265; HBD: 9; MBD: 417; Fig. 1A–C), substantially outperforming multi-GWAS. For SBD, RBD, WBD, and MBD, meta-GWAS significant SNPs encompassed all multi-GWAS hits, with partial overlap for HBD (five shared SNPs; Table 3). Genomic inflation factors ($$\lambda\,$$$$=$$ 1.014–1.026) indicated minimal population stratification bias (Fig. S2). The leading SNP, rs343467711 (SSC1:160,174,493), showed the strongest association with WBD (*P* $$=$$ 1.20 $$\times$$ 10^–11^, meta-GWAS; *P* $$=$$ 1.45 $$\times$$ 10^–8^, multi-GWAS), while rs342950505 (SSC1:161,123,588) was most significant for SBD, RBD, HBD, and MBD (*P* $$=$$ 1.65 $$\times$$ 10^–9^ to 2.06 $$\times$$ 10^–14^, meta-GWAS; *P* $$=$$ 2.69 $$\times$$ 10^–9^ to 5.74 $$\times$$ 10^–13^, multi-GWAS; Fig. 1A).

**Table 3:** SNP identification in different backfat thickness traits using multi-GWAS and meta-GWAS

Trait	Multi-GWAS (Significant^1^/Suggestive^2^ SNP)	Meta-GWAS (Significant^1^/Suggestive^2^ SNP)	Overlap
SBD	14 (1/13)	48 (1/43)	1
RBD	274 (63/211)	1,106 (421/685)	63
WBD	17 (1/16)	735 (265/470)	1
HBD	65 (6/59)	55 (9/46)	5
MBD	261 (46/215)	830 (417/413)	46

*SBD* Shoulder backfat depth, *RBD* 6^th^–7^th^ rib backfat depth, *WBD* Waist backfat depth, *HBD* Hip backfat depth, *MBD* Mean of backfat depth

^1^Significant SNPs: *P* $$<$$ 5 $$\times$$ 10^-8^

^2^Suggestive SNPs: *P* $$<$$ 1 $$\times$$ 10^-8^

Given the broad QTL intervals from GWAS, functional validation is critical for pinpointing causal genes regulating BFT across pig body regions and their mechanisms. As robust causal variant hypotheses are a prerequisite for validation, we used heuristic fine-mapping techniques [46], integrating FINEMAP [45] and SuSiEx [21], to refine QTLs and improve variant identification accuracy. The summary results from the multi-GWAS ($$n$$$$=$$ 3,578) were utilized to identify candidate genomic regions, which were defined based on the most significant loci from five BFT phenotypes (SBD, RBD, WBD, HBD, and MBD) and their flanking regions (LD $$>$$ 0.8). These regions were ranked according to GWAS *P*-values and LD structure, revealing a shared interval on SSC1:159,931,751–161,583,240 (1.65 Mb). Within this interval, FINEMAP was programmed to estimate one to four causal variants, prioritizing candidates based on posterior inclusion probability (PIP) and LD structure [68]. The final selection region SSC1:160,883,673–161,514,225 (630.6 kb) included 14 SNPs with PIP $$\ge$$ 0.5 (Fig. 1D). SuSiEx validated the top PIP SNPs for SBD, RBD, WBD, and HBD, accounting for population heterogeneity, and aligned with meta-GWAS and multi-GWAS findings (Fig. 1E). Although rs343467711 fell outside this interval, it remained the primary SNP for MBD. Incorporating SNPs with LD $$r^2$$ $$>$$ 0.8 and *P *$$<$$ 5 $$\times$$ 10^–8^ (meta-GWAS) or* P *$$<$$ 1 $$\times$$ 10^–6^ (multi-GWAS), we identified 34 candidate causal variants within the refined 630.6-kb region. In conclusion, we identified a major QTL on SSC1 (160.9–161.5 Mb) significantly associated with BFT across five traits in pigs. Fine-mapping prioritized 34 candidate causal variants, including rs342950505, that are likely to underlie region-specific fat deposition.

### Integrating epigenomics to identify tissue-specific variants and predict SAD

**Fig. 2:**
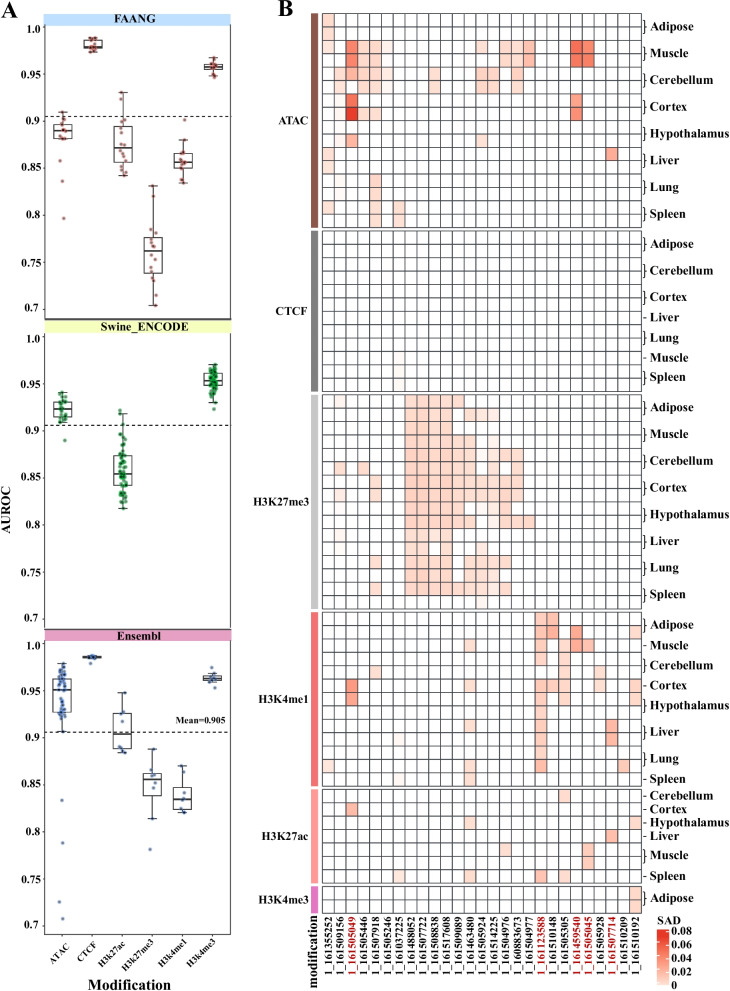
Epigenetic modification analysis and tissue-specific variant ranking for candidate causal variants. **A** Boxplot of AUROC values for epigenetic modifications across FAANG, Swine ENCODE, and Ensembl datasets, with a mean AUROC of 0.905 across all data. **B** Heatmap of the top 20 tissues ranked by SAD values for candidate causal variants across datasets, with FAANG-represented tissue highlighted. The red highlighted variants, include 1_161123588 (rs342950505), 1_161505049 (rs322437579), 1_161459540 (rs1111533281), 1_161505045 (rs344993192), and 1_161507714 (rs336148953), which are key candidate variants

To explore the tissue-specific regulatory roles of the 34 candidate variants identified through fine-mapping, we employed *Basenji* [65], a computational approach that prioritizes regulatory variants. Leveraging three multi-tissue epigenetic datasets—FAANG [63], Swine ENCODE [64], and Ensembl—*Basenji* was trained to predict chromatin accessibility across various pig tissues. The model achieved a mean area under the receiver operating characteristic curve (AUROC) of 0.905, with CTCF binding predictions reaching a peak AUROC of 0.988 and H3K27me3 predictions at a minimum of 0.704 in the FAANG dataset (Fig. 2A).

To evaluate the predictive performance of our model, we assessed its accuracy using the well-characterized *IGF2* locus on SSC2, which contains a known causal variant influencing muscle development [69]. Across three muscle-specific epigenetic datasets, our model accurately identified the causal variant SSC2_1483817, in which the C allele enhances CTCF binding compared to the T allele (Fig. S8C), consistent with previous functional studies. These findings confirm the model’s capability to detect tissue-specific regulatory effects. Subsequently, we applied *Basenji* to predict activity differences for the 34 candidate regulatory variants using models trained on the aforementioned datasets (Table S2). We focused on five of the top 20 SNP Activity Differences (SAD) (Fig. 2B).

In the FAANG dataset, the noncoding variant rs342950505 (1:161,123,588) has consistently high H3K4me1 signals across different tissues, most notably in adipose tissue (SAD $=$ 0.0134), indicating a broad-acting enhancer. At rs322437579 (1:161,505,049), cortex-specific signals from ATAC-seq (SAD $=$ 0.070), H3K4me1 (SAD $=$ 0.033), and H3K27ac (SAD $=$ 0.018) indicate enhancer activity. Similarly, at rs1111533281 (1:161,459,540) and rs344993192 (1:161,505,045), muscle-specific ATAC-seq (SAD $=$ 0.051 and 0.045, respectively) and H3K4me1/H3K27ac signals support enhancer roles. At rs336148953 (1:161,507,714), liver-specific ATAC-seq (SAD $=$ 0.025) and H3K4me1/H3K27ac signals support enhancer activity more strongly. Variants associated with H3K27me3 silencing, such as rs328702901 (1:161,517,608), were deprioritized (Fig. 2B, Figs. S3 and S4). In parallel, summary statistics from the PigBiobank GWAS [70] revealed significant associations across multiple BFT traits (M_BFT, D_BFT, Y_BFT, S_BFT_100, and S_BFT_115). Notably, rs342950505 showed consistently strong association signals, whereas neighboring SNPs exhibited markedly lower significance—corroborating our GWAS, fine-mapping, and *Basenji*-based predictions (Fig. S5). Accordingly, we propose that rs342950505 functions as a general enhancer across multiple tissues, while rs322437579, rs1111533281, rs344993192, and rs336148953 function as tissue-specific enhancers in the cortex, muscle, and liver, respectively. Collectively, these variants are likely to modulate BFT by influencing neural signaling and lipid metabolism.

### Multi-omics converges on candidate causal genes, tissues, and cell types

**Fig. 3:**
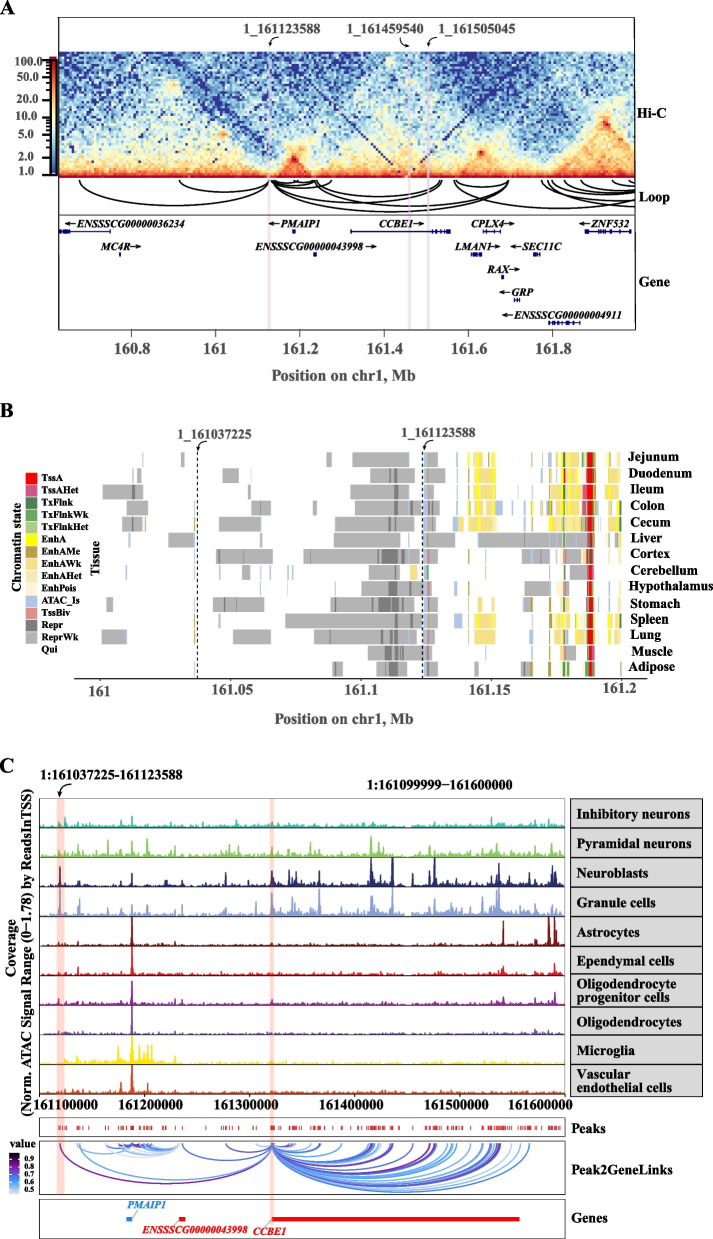
Multi-omics chromatin and single-cell regulatory landscapes of the BFT locus. **A** Higher-order chromatin interactions in backfat tissue identified via Hi-C analysis (SSC1:160,800,000–162,000,000), binned at 5 kb resolution. **B** Chromatin state annotation of the 86.363 kb backfat thickness locus across 14 pig tissues, illustrating 15 distinct chromatin states. These include promoters (TssA, TssAHet, TssBiv), TSS-proximal transcribed regions (TxFlnk, TxFlnkWk, TxFlnkHet), enhancers (EnhA, EnhAMe, EnhAWk, EnhAHet, EnhPois), repressor regions (Repr, ReprWk), quiescent regions (Qui), and ATAC islands. **C** Single-cell ATAC-seq (scATAC-seq) results for the brain (SSC1:161,099,999–161,600,000), highlighting Peak2GeneLinks across 10 distinct brain cell types

To further investigate downstream regulatory targets, fine-mapping of the broad SSC1:160,883,673–161,514,225 (630.6 kb) locus revealed multiple candidate genes, though the regulatory target of the strong candidate SNP rs342950505 remained initially uncharacterized. To refine target gene prediction, we performed high-throughput chromosome conformation capture (Hi-C) analysis in adipose tissue across the locus, identifying topologically associating domains (TADs) and chromatin loops. This analysis found interactions between rs342950505 (1:161,123,588) and the promoter regions of *ENSSSCG00000036234*, *ENSSSCG00000043998*, *MC4R*, *PMAIP1*, *CCBE1*, *LMAN1*, *CPLX4*, and *RAX*, implying that they are potential target genes of this regulatory variant (Fig. 3A).

**Fig. 4:**
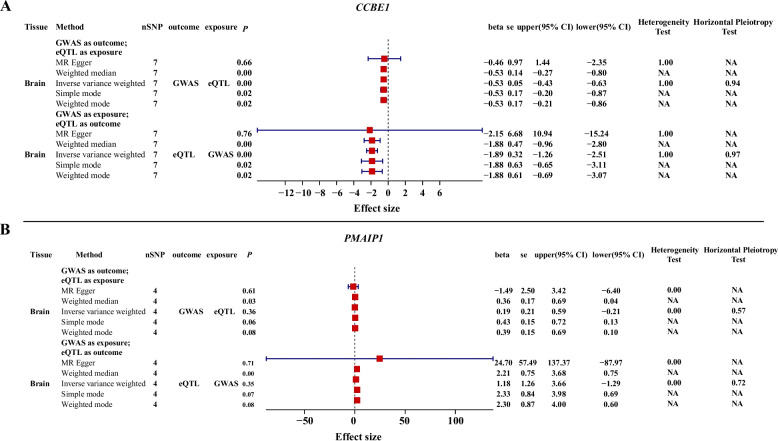
Bi-directional MR analysis between MBD and brain eQTLs. **A** Bi-directional MR analysis between mean backfat depth (MBD) and *CCBE1* eQTL in the brain based on PigGTEx eQTL data. **B** Bi-directional MR analysis between MBD and *PMAIP1* eQTL in the brain based on PigGTEx eQTL data. Results are presented as effect sizes (beta) with 95% confidence intervals. nSNP indicates the number of instrumental SNPs used in each test

To elucidate the regulatory mechanisms of this variant, a 15-state chromatin model across 14 tissues identified an enhancer-associated peak spanning SSC1:161,118,800–161,121,600 in the cerebellum (Fig. 3B). Therefore, we conducted single-cell ATAC-seq (scATAC-seq) within the SSC1:161,099,999–161,600,000 brain region. A peak containing rs342950505 was associated with *PMAIP1* expression in inhibitory neurons via a peak-to-gene relationship, indicating cell-type-specific transcriptional regulation (Fig. 3C). This interaction likely mediates hypothalamic regulation of appetite and feeding behavior through neural pathways, contributing to BFT variation [71, 72]. Single-cell ATAC-seq also identified a chromatin accessibility peak near SSC1:161,118,800–161,121,600 in neuroblasts and granule cells, implicating *CCBE1* regulation by this peak region (Fig. 3C). These findings support a model in which *CCBE1* modulates BFT through neural control of energy balance. To validate gene–trait associations, we integrated multi-cohort GWAS data with PigGTEx [55] molecular QTL colocalization (PP4 $>$ 0.5) [57, 73], two-sample MR, TWAS, and MAGMA. MR analyses revealed significant associations between gene expression and BFT in the brain. All five different MR methods showed a negative bidirectional relationship between *CCBE1* expression and BFT (*CCBE1*$\to$ BFT, $\beta$ $\le$ −2.15, BFT $\to$* CCBE1*, $\beta$ $\le$ −0.53). Sensitivity analyses also confirmed the robustness of this result (heterogeneity *P* $>$ 0.5; multiplicity *P* $\ge$ 0.1) (Fig. 4A). Similarly, with the exception of the MR-Egger results, *PMAIP1* expression was found to have a bidirectional positive correlation with BFT (*PMAIP1*$\to$ BFT, $\beta$ $\ge\,$ 1.18, BFT $\to$*PMAIP1*, $\beta$ $\ge$ 0.19) (Fig. 4B). TWAS identified multiple BFT-associated genes (*P* $<$ 1 $\times$ 10^–3^) [74] across 20 tissues, including a significant *PMAIP1*–BFT association in the hypothalamus (WBD: $Z$ $=$ 3.68, *P *$=$ 2.33 $\times$ 10^–4^; Fig. S6A). MAGMA further implicated *PMAIP1* (RBD, *P *$=$ 0.16) and *CCBE1* (MBD,* P *$=$ 2.85 $\times$ 10^–5^) as key contributors (Fig. S6B). Collectively, these results highlight *PMAIP1* and *CCBE1* as key genes of BFT via brain-mediated mechanisms. Additionally, *SEC11C* and *ENSSSCG00000004911* showed colocalization signals in muscle and adipose tissues, with the latter, linked to lipid metabolism, exhibiting TWAS association in adipose tissue (RBD, $Z$ $=$ 4.11, *P *$=$ 3.95 $\times$ 10^–5^; Fig. S7A–D).

Together, our results support a multifaceted regulatory model for BFT, in which (1) a peak harboring rs342950505 modulates *PMAIP1* expression in hypothalamic inhibitory neurons, affecting energy homeostasis, and (2) an adjacent peak regulates *CCBE1* in neuroblasts and granule cells, potentially modulating brain-mediated signaling pathways. These central mechanisms likely operate in concert with peripheral regulatory effects mediated by *SEC11C* and *ENSSSCG00000004911* in muscle and adipose tissues.

### Mechanistic insights of non-coding BFT variants

**Fig. 5:**
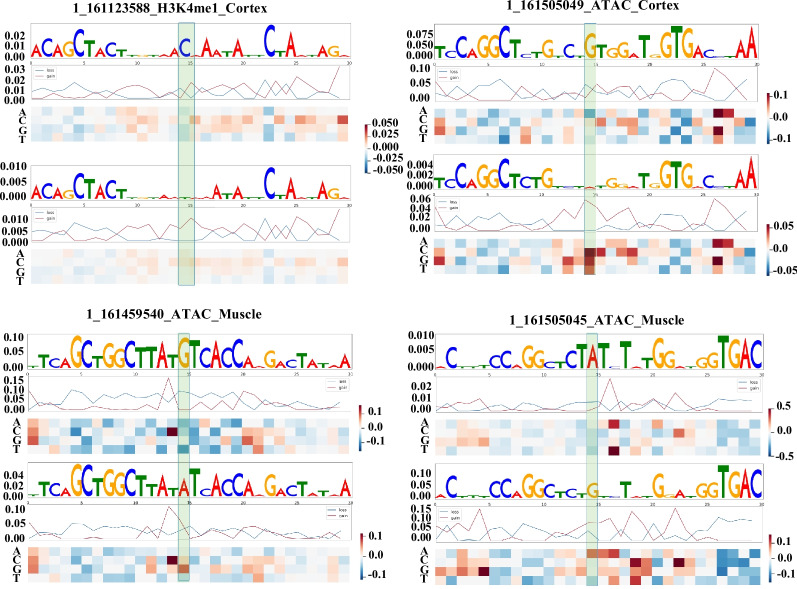
In silico saturation mutagenesis analysis of candidate variants. 1_161123588 (rs342950505) in cortex; 1_161505049 (rs322437579) in cortex; 1_161459540 (rs1111533281) in muscle; 1_161505045 (rs344993192) in muscle

Given the noncoding nature of the identified variants, we trained an epigenetic convolutional neural network model to integrate large-scale chromatin accessibility and histone modification datasets to learn regulatory patterns and to prioritize candidate tissues including muscle, brain, intestine, and liver. This approach identified 34 candidate causal variants, including five core-associated SNPs [23, 26]. To functionally characterize these variants, we performed in silico saturation mutagenesis by systematically substituting nucleotides within a 30-bp window centered on rs342950505, rs322437579, rs1111533281, rs344993192, and rs336148953, and evaluating their predicted impact on chromatin features [65]. Notably, at rs342950505 (1:161,123,588), the C allele increased H3K4me1 levels relative to the T allele in the cortex (SAD $=$ 0.0114; Fig. 5), with a similar elevation also observed in adipose tissue (SAD $=$ 0.0134; Fig. S8A). At rs322437579 (1:161,505,049), the G allele enhanced chromatin accessibility compared to the A allele (Fig. 5). Similar regulatory effects were observed for rs1111533281 (1:161,459,540), rs344993192 (1:161,505,045), and rs336148953 (1:161,507,714) (Fig. 5; Fig. S8A and B). This integrative framework identified five high-confidence regulatory variants as potential molecular targets for modulating fat deposition phenotypes.

We propose that rs342950505, rs322437579, rs1111533281, rs344993192, and rs336148953 function as distal enhancers regulating gene expression in adipose tissue, cortex, muscle, and liver, respectively, contributing to increased BFT through coordinated lipid metabolism and brain-mediated signaling pathways.

## Discussion

### Genetic architecture of backfat thickness on SSC1 in pigs

Previous GWAS of pig BFT, largely based on SNP arrays and imputation, identified broad QTLs but lacked resolution to pinpoint effector genes, relevant tissues, or causal variants [6, 18, 70, 75–79]. Here, leveraging chip-imputed and whole-genome sequencing (WGS) data from 3,578 pigs, we refined a major SSC1 QTL to a 630.6-kb interval (160,883,673–161,514,225), identifying 34 candidate causal SNPs with higher resolution than previous studies. For instance, Gozalo-Marcilla et al. [80] mapped this region to 4 Mb, while others reported even broader intervals [2, 77]. Using a sequence-based deep convolutional neural network *Basenji* model, we predicted five enhancer variants across metabolic tissues, including adipose, cortex, muscle, and liver. Integrative analyses (Hi-C, tissue chromatin state annotations, scATAC-seq, MR, TWAS) converged on *PMAIP1* and *CCBE1* as key target genes: a peak harboring rs342950505 modulates *PMAIP1* expression in inhibitory neurons, implicating hypothalamic regulation; a peak at a nearby locus (SSC1:161.1188–161.1216 Mb) was associated with *CCBE1* expression activity in neuroblasts and granule cells, supporting a brain regulatory mechanism. Additional candidate genes, *ENSSSCG00000004911* and *SEC11C*, were supported by molQTL colocalization, MR, TWAS, and MAGMA.

### Functional insights into candidate casual genes

Functional analyses identified *PMAIP1, CCBE1, ENSSSCG00000004911*, and *SEC11C* as key regulators of BFT. We characterized the adipose-related functions of *PMAIP1*—the strongest target gene of BFT association. *PMAIP1*, a pro-apoptotic Bcl-2 family member, drives mitochondrial apoptosis by antagonizing MCL1, releasing cytochrome c, and activating caspases. Its dysfunction in brain tissues disrupts hypothalamic neuroendocrine circuits that regulate metabolism. Human GWAS have linked *PMAIP1* to body mass index [81, 82], visceral adiposity, waist–hip ratio [83], apolipoprotein A1 levels [84], and various brain-related measurements [85], suggesting that it may be a therapeutic target for metabolic disorders [86]. In pigs, genetic associations link *PMAIP1* to average daily gain (ADG) [16, 87], subcutaneous adipose thickness [88], body weight [89], and BFT [90]. Our MR and TWAS analyses highlight the brain and hypothalamus as a primary region, with Hi-C identifying a chromatin interaction at rs342950505 and scATAC-seq placing this SNP in an active regulatory peak in inhibitory neurons, supporting *PMAIP1* as the target gene. We propose that neuronal *PMAIP1* affects hypothalamic mitochondrial function and leptin signaling, with variation at rs342950505 impacting fat deposition—a *Basenji*-predicted enhancer whose activity awaits experimental validation.

*CCBE1*, involved in extracellular matrix remodeling and VEGF signaling [91, 92], is tied to human body height [93] and thyroid-stimulating hormone levels [94]. In pigs, previous GWAS by Ding et al. [79], Zhu et al. [95], Zhang et al. [96] and Zeng et al. [77] have consistently associated the SSC1:161,118,800–161,121,600 region with BFT across multiple breeds and annotated it for roles in fat metabolism. Our multi-omics data suggest neuronal *CCBE1* regulation links central nervous system expression to peripheral adiposity, though the exact causal variants remain to be mapped.

*SEC11C*, a homolog of *SEC11* essential for signal peptide processing, modulates BFT through its role in muscle protein trafficking [97], as supported by multi-omics prioritization, though its mechanistic details require further exploration. A meta-GWAS identified a QTL at SSC1:155.98–162.19 Mb, encompassing *ENSSSCG00000004911*, associated with BFT and growth traits in Duroc, Landrace, and Yorkshire pigs [77], and overlapping with lipid metabolism QTLs [96, 98]. PigGTEx [55] eQTL-based MR, TWAS, and MAGMA analyses suggest regulatory activity in adipose tissue (Fig. S9). Collectively, these genes contribute to a regulatory network integrating neuronal and adipose signaling pathways to governing fat deposition in pigs.

### Limitations and future directions

There are limitations to our study. Although we identified candidate effector tissues, genes, and regulatory variants, their precise mechanisms of action are still unclear and require functional validation. For example, the transcription factor(s) binding to the candidate causal variant rs342950505 remains unknown. Identification of such factors will be crucial for elucidating its regulatory function. Experimental approaches—including cell type–specific chromatin conformation capture, RNA interference, enhancer–reporter assays, scRNA-seq/ATAC-seq, CRISPR/Cas9 editing, lipid profiling, and CUT&RUN—will be essential to validate the regulatory mechanisms of this variant.

Our *Basenji* model, which was trained on multi-tissue epigenomic datasets, effectively captures broad regulatory landscapes and accurately predicts enhancers. Future iterations of the model, trained on single-cell-type-specific ATAC-seq datasets or other epigenomic data, could further elucidate the genetic architecture of BFT and improve predictive accuracy. Furthermore, weaker-than-expected colocalization between GWAS and molQTLs points to statistical restrictions, tissue-specific marker deficits, or multiple causative variants [99, 100]. Enhanced genetic diversity, uniform phenotyping, and larger molQTL datasets from GWAS-matched tissues are all essential for improving statistical power.

## Conclusion

In conclusion, our integrative fine-mapping of a significant SSC1 QTL prioritizes five candidate causal variants and, through multi-omics data integration, converges on *PMAIP1*, *CCBE1*, *ENSSSCG00000004911*, and *SEC11C* as candidate causal genes for BFT, alongside regulatory effects in three brain-related cell types. These findings enhance our understanding of the genetic architecture of BFT and offer insights relevant to cross-species metabolic studies.

## Supplementary Information

Additional file 1: Table S1. Sources of epigenetic BED files. Table S2. Multi-GWAS summary of candidate causal variants. Table S3. Meta-GWAS summary of candidate causal variants.

Additional file 2: Fig. S1. Manhattan plot of multi-GWAS for five backfat thickness (SBD, shoulder backfat depth; RBD, 6^th^–7^th^ rib backfat depth; WBD, waist backfat depth; HBD, hip backfat depth; MBD, mean backfat depth) on SSC1. Fig. S2. Q-Q plot of SNP effects from meta-GWAS and multi-GWAS. Fig. S3. Top 20 tissues by SAD values for candidate variants (Swine ENCODE). The red highlighted variants include 1_161505049 (rs322437579), 1_161505045 (rs344993192), and 1_161507714 (rs336148953). Fig. S4. Top 20 tissues by SAD values for candidate variants (Ensembl). The red highlighted variants include 1_161123588 (rs342950505), 1_161505049 (rs322437579), and 1_161505045 (rs344993192). Fig. S5. Manhattan plot of BFT traits from the PigBioBank dataset. Green dots represent 34 candidate causal variants; red dots indicate four core candidate causal variants. Fig. S6. eQTL-TWAS and gene-set analysis plots. A, eQTL-TWAS plot depicting associations between multi-GWAS traits and various tissues. B, Manhattan plot of gene-set analysis derived from meta-GWAS results, where each dot represents the starting position of a gene, and distinct dots correspond to different traits. Fig. S7. Colocalization plots of multi-GWAS with tissue-specific eQTLs. A, HBD with adipose eQTL; B, WBD with muscle eQTL; C, RBD with muscle eQTL; D, MBD with adipose eQTL. HBD (hip backfat depth), WBD (waist backfat depth), RBD (6^th^–7^th^ rib backfat depth), and the mean backfat depth (MBD) are shown. Fig. S8. In silico saturation mutagenesis analysis of candidate variants. A, 1_161123588 (rs342950505) in adipose; B, 1_161507714 (rs336148953) in liver; C, SSC2_1483817 in muscle. Fig. S9. Bi-directional MR analysis between mean backfat depth (MBD) and adipose eQTLs. Results are presented as effect sizeswith 95% confidence intervals. nSNP indicates the number of instrumental SNPs used in each test.

## Data Availability

The GWAS summary statistics generated in this study have been deposited in the NGDC GWAS Atlas under the BioProject accession number PRJCA041393, and are publicly available at: https://ngdc.cncb.ac.cn/bioproject/.

## Abbreviations

ATAC-seq: Assay transposase accessible chromatin with sequencing
BFT: Backfat thickness
Chip-seq: Chromatin immunoprecipitation followed by Sequencing
CNN: Convolutional neural network
DLY: Duroc $\times$ Landrace $\times$ Yorkshire hybrid
GWAS: Genome-wide association study
HBD: Hip backfat depth
Hi-C: High-throughput chromosome conformation capture
LR: Landrace
LY: Landrace and Yorkshire hybrid
MBD: Mean backfat depth
meta-GWAS: Meta-analysis of GWAS
molQTL: Molecular quantitative trait loci
MR: Mendelian randomization
multi-GWAS: Two-population combined GWAS
QTL: Quantitative trait locus
RBD: 6^th^–7^th^ rib backfat depth
SBD: Shoulder backfat depth
scATAC-seq: Single-cell ATAC sequencing
SNP: Single nucleotide polymorphism
SSC: *Sus scrofa* Chromosome
TWAS: Transcriptome-wide association study
WBD: Waist backfat depth
WGS: Whole-genome sequencing
YK: Yorkshire
