# Supplementary material for: Multi-omics integration reveals Chr1 associated QTL mediating backfat thickness in pigs
Source: J Anim Sci Biotechnol. 2025 Oct 20;16:133. doi: 10.1186/s40104-025-01254-1 (PMC12536534; doi:10.1186/s40104-025-01254-1)
Supplement: Supplementary file 2 — Additional file 2: Fig. S1. Manhattan plot of multi-GWAS for five backfat thickness (SBD, shoulder backfat depth; RBD, 6th–7th rib backfat depth; WBD, waist backfat depth; HBD, hip backfat depth; MBD, mean backfat depth) on SSC1. Fig. S2. Q-Q plot of SNP effects from meta-GWAS and multi-GWAS. Fig. S3. Top 20 tissues by SAD values for candidate variants (Swine ENCODE). The red highlighted variants include 1_161505049 (rs322437579), 1_161505045 (rs344993192), and 1_161507714 (rs336148953). Fig. S4. Top 20 tissues by SAD values for candidate variants (Ensembl). The red highlighted variants include 1_161123588 (rs342950505), 1_161505049 (rs322437579), and 1_161505045 (rs344993192). Fig. S5. Manhattan plot of BFT traits from the PigBioBank dataset. Green dots represent 34 candidate causal variants; red dots indicate four core candidate causal variants. Fig. S6. eQTL-TWAS and gene-set analysis plots. A, eQTL-TWAS plot depicting associations between multi-GWAS traits and various tissues. B, Manhattan plot of gene-set analysis derived from meta-GWAS results, where each dot represents the starting position of a gene, and distinct dots correspond to different traits. Fig. S7. Colocalization plots of multi-GWAS with tissue-specific eQTLs. A, HBD with adipose eQTL; B, WBD with muscle eQTL; C, RBD with muscle eQTL; D, MBD with adipose eQTL. HBD (hip backfat depth), WBD (waist backfat depth), RBD (6th–7th rib backfat depth), and the mean backfat depth (MBD) are shown. Fig. S8. In silico saturation mutagenesis analysis of candidate variants. A, 1_161123588 (rs342950505) in adipose; B, 1_161507714 (rs336148953) in liver; C, SSC2_1483817 in muscle. Fig. S9. Bi-directional MR analysis between mean backfat depth (MBD) and adipose eQTLs. Results are presented as effect sizeswith 95% confidence intervals. nSNP indicates the number of instrumental SNPs used in each test. [file 40104_2025_1254_MOESM2_ESM.docx]

# **Supplementary Figures**

|  |
| --- |
| Fig. S1. Manhattan plot of multi-GWAS for five backfat thickness (SBD, shoulder backfat depth; RBD, 6th–7th rib backfat depth; WBD, waist backfat depth; HBD, hip backfat depth; MBD, mean backfat depth) on SSC1. |

| 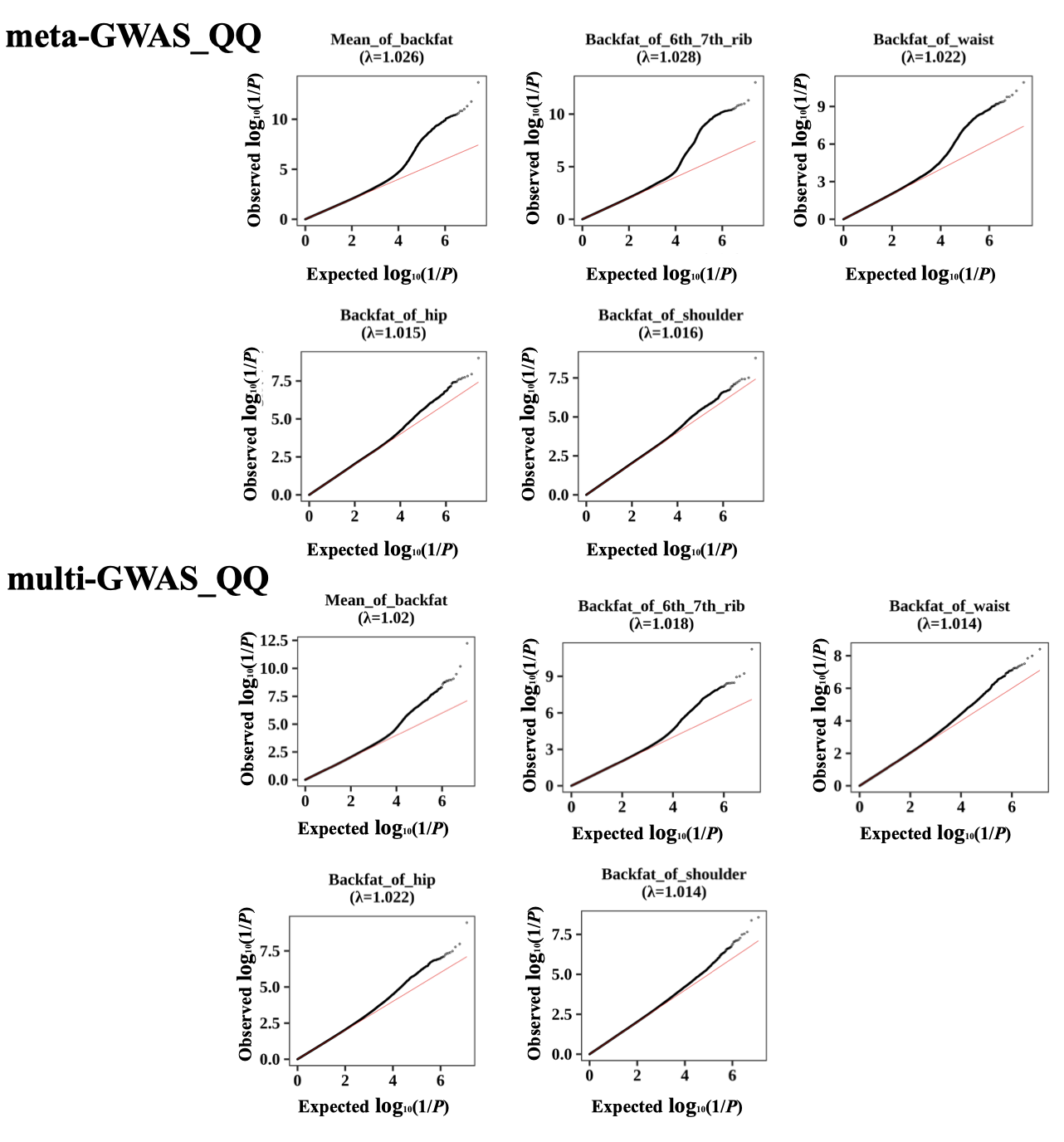 |
| --- |
| **Fig. S2.** Q-Q plot of SNP effects from meta-GWAS and multi-GWAS. |

|  |
| --- |
| Fig. S3. Top 20 tissues by SAD values for candidate variants (Swine ENCODE). The red highlighted variants include 1_161505049 (rs322437579), 1_161505045 (rs344993192), and 1_161507714 (rs336148953). |

|  |
| --- |
| Fig. S4. Top 20 tissues by SAD values for candidate variants (Ensembl). The red highlighted variants include 1_161123588 (rs342950505), 1_161505049 (rs322437579), and 1_161505045 (rs344993192). |

| 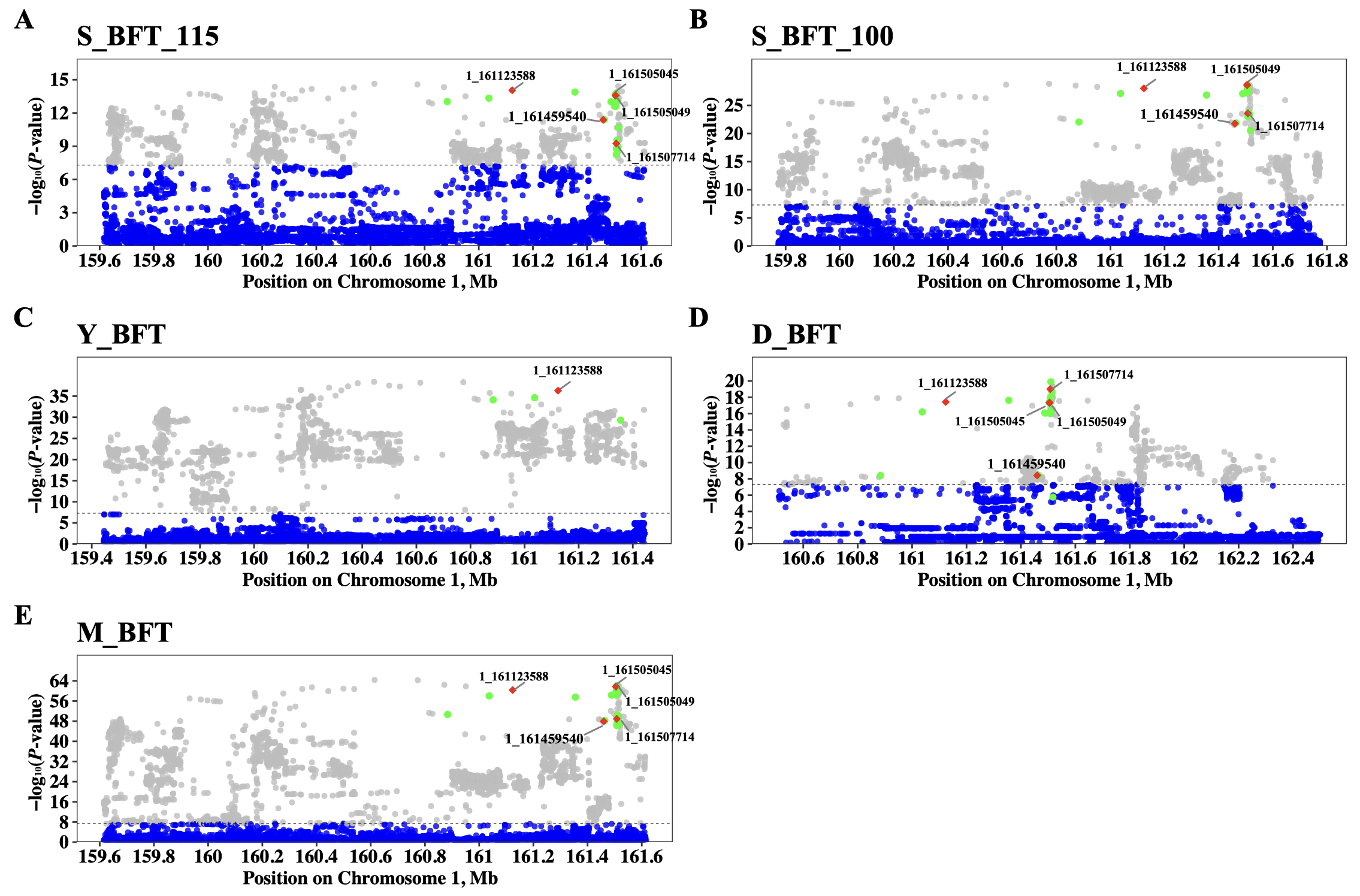 |
| --- |
| **Fig. S5.** Manhattan plot of BFT traits from the PigBioBank dataset. Green dots represent 34 candidate causal variants; red dots indicate four core candidate causal variants. |

|  |
| --- |
| **Fig. S6.** eQTL-TWAS and gene-set analysis plots. A, eQTL-TWAS plot depicting associations between multi-GWAS traits and various tissues. B, Manhattan plot of gene-set analysis derived from meta-GWAS results, where each dot represents the starting position of a gene, and distinct dots correspond to different traits. |

| 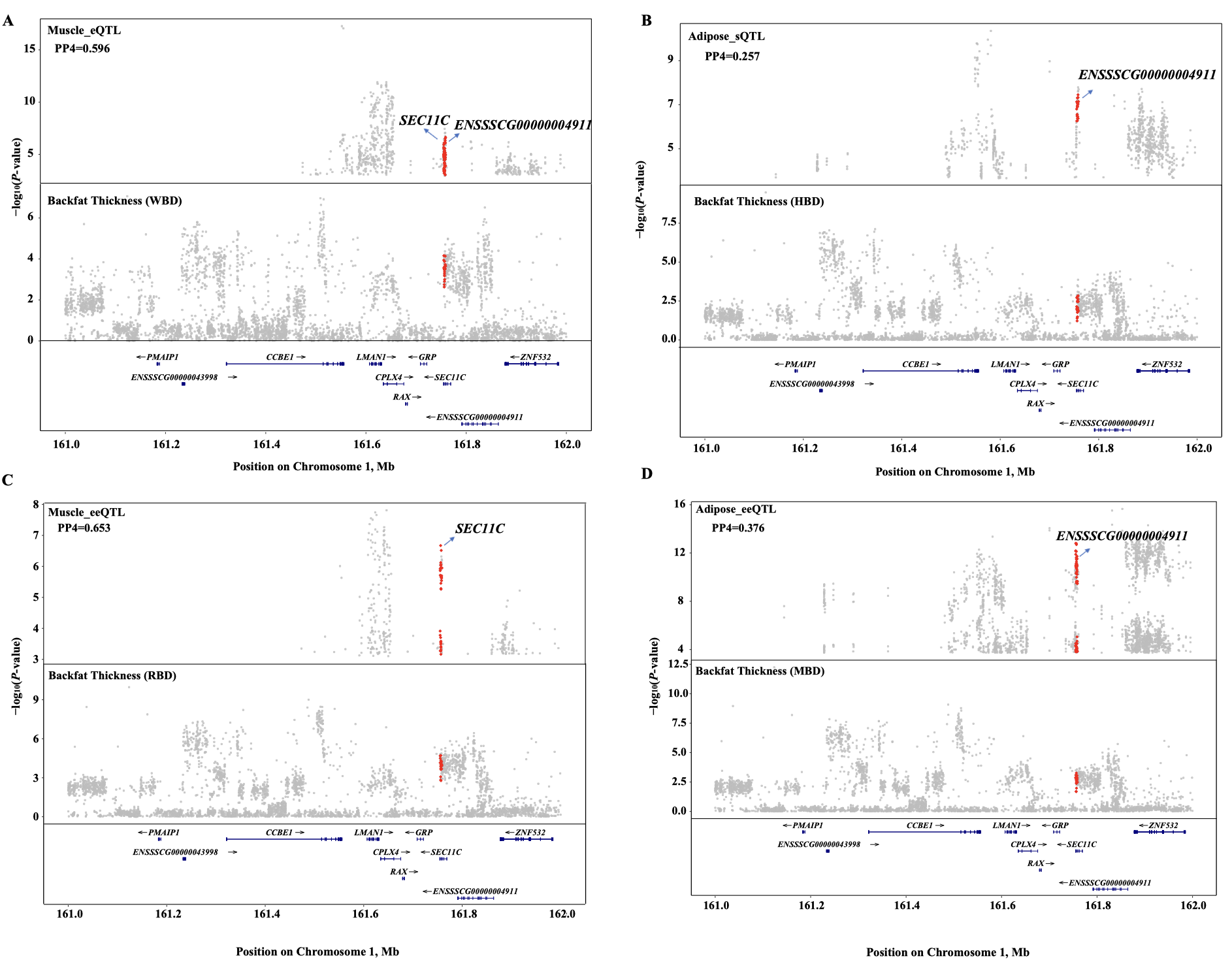 |
| --- |
| **Fig. S7.** Colocalization plots of multi-GWAS with tissue-specific eQTLs. A, HBD with adipose eQTL; B, WBD with muscle eQTL; C, RBD with muscle eQTL; D, MBD with adipose eQTL. HBD (hip backfat depth), WBD (waist backfat depth), RBD (6th–7th rib backfat depth), and the mean backfat depth (MBD) are shown. |

| 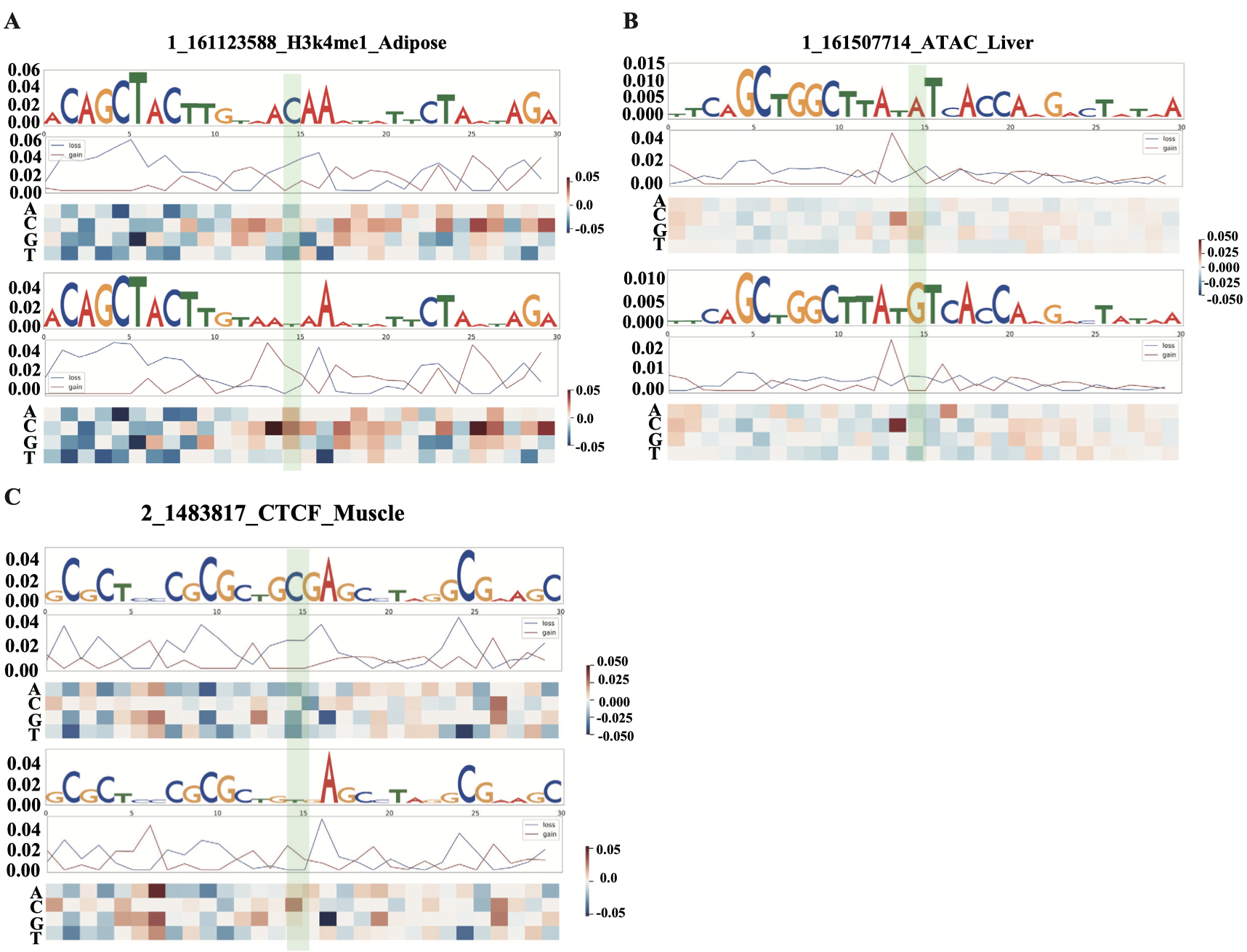 |
| --- |
| **Fig. S8.** In *silico* saturation mutagenesis analysis of candidate variants. A, 1_161123588 (rs342950505) in adipose; B, 1_161507714 (rs336148953) in liver; C, SSC2_1483817 in muscle. |

| 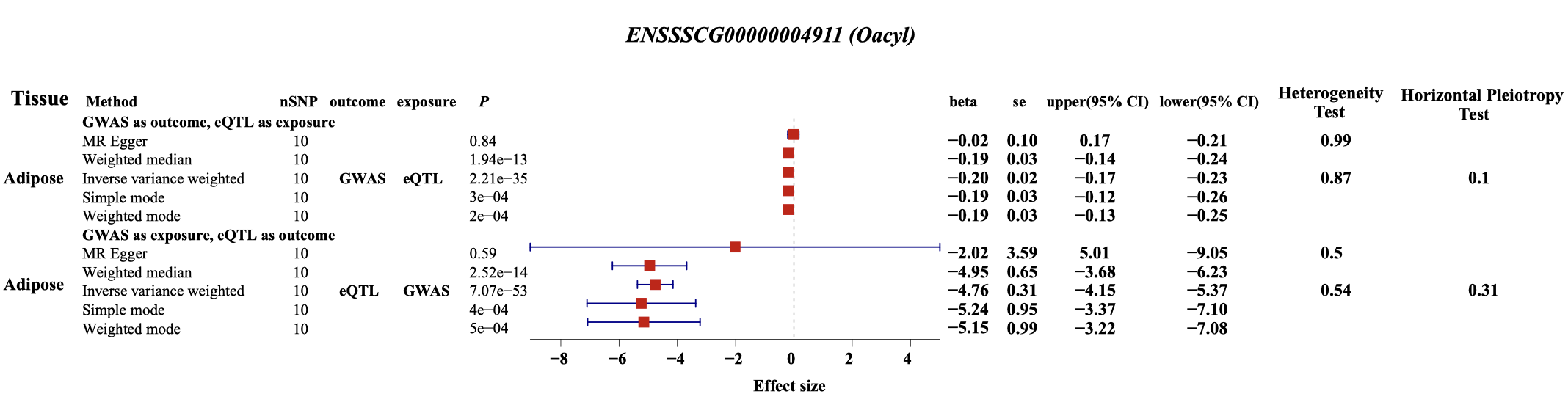 |
| --- |
| **Fig. S9.** Bi-directional MR analysis between mean backfat depth (MBD) and adipose eQTLs. Results are presented as effect sizes (beta) with 95% confidence intervals. nSNP indicates the number of instrumental SNPs used in each test. |
